# Supplementary material for: Humanizing the yeast origin recognition complex
Source: Nat Commun. 2021 Jan 4;12:33. doi: 10.1038/s41467-020-20277-y (PMC7782691; doi:10.1038/s41467-020-20277-y)
Supplement: Supplementary file 1 — Supplementary Information [file 41467_2020_20277_MOESM1_ESM.pdf]

Supplementary Information:

Humanizing the Yeast Origin Recognition Complex

a

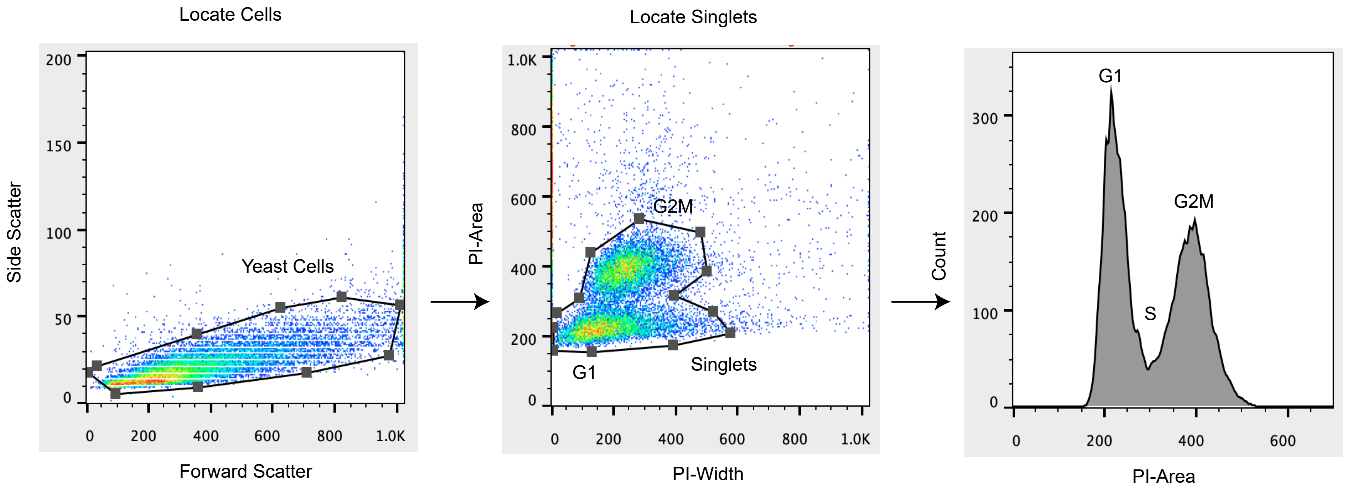

b

Cell state	WT G1	WT S	WT G2M	Orc4-IHΔ G1	Orc4-IHΔ S	Orc4-IHΔ G2M
	Percentage of yeast cell population (%)					
	WT			Orc4-IHΔ		
Cell state	G1	S	G2M	G1	S	G2M
Asynchronous	44	10.5	39.7	25.9	5.12	64.3
G1 arrest	> 90	/	/	78.9	8.53	9.83
15 mins post release	> 90	/	/	80.5	9.01	7.3
30 mins post release	> 85	/	/	> 85	/	/
45 mins post release	Migration from G1 to S					
60 mins post release	8.55	8.15	77.2	13.2	10.4	61.6
75 mins post release	7.4	7.84	79.6	13.3	9.36	63.1
90 mins post release	7.45	11.1	72.3	14.3	6.89	69.9
105 mins post release	26.6	8.7	59.1	14.5	9.94	65.2
120 mins post release	43	7.69	46.8	16.2	7.03	69.9

Supplementary Fig 1. Gating strategies and percentage of cells in each population of FACS.

a. Figure illustrating the sequential gating strategies used in FACS analysis. Standard FSC/SSC gate was applied to starting population, followed by PI-Area against PI-Width gate to identify PI -stained singlets.

b. Table showing percentage of G1, S or G2M phase cells (derived from FACS analysis in Fig. 2c) in WT and Orc4-IHΔ cells at asynchronous culture, G1 arrest, and 15 minute intervals post release from G1 arrest, alpha factor was added back at 75 minute post G1 release.

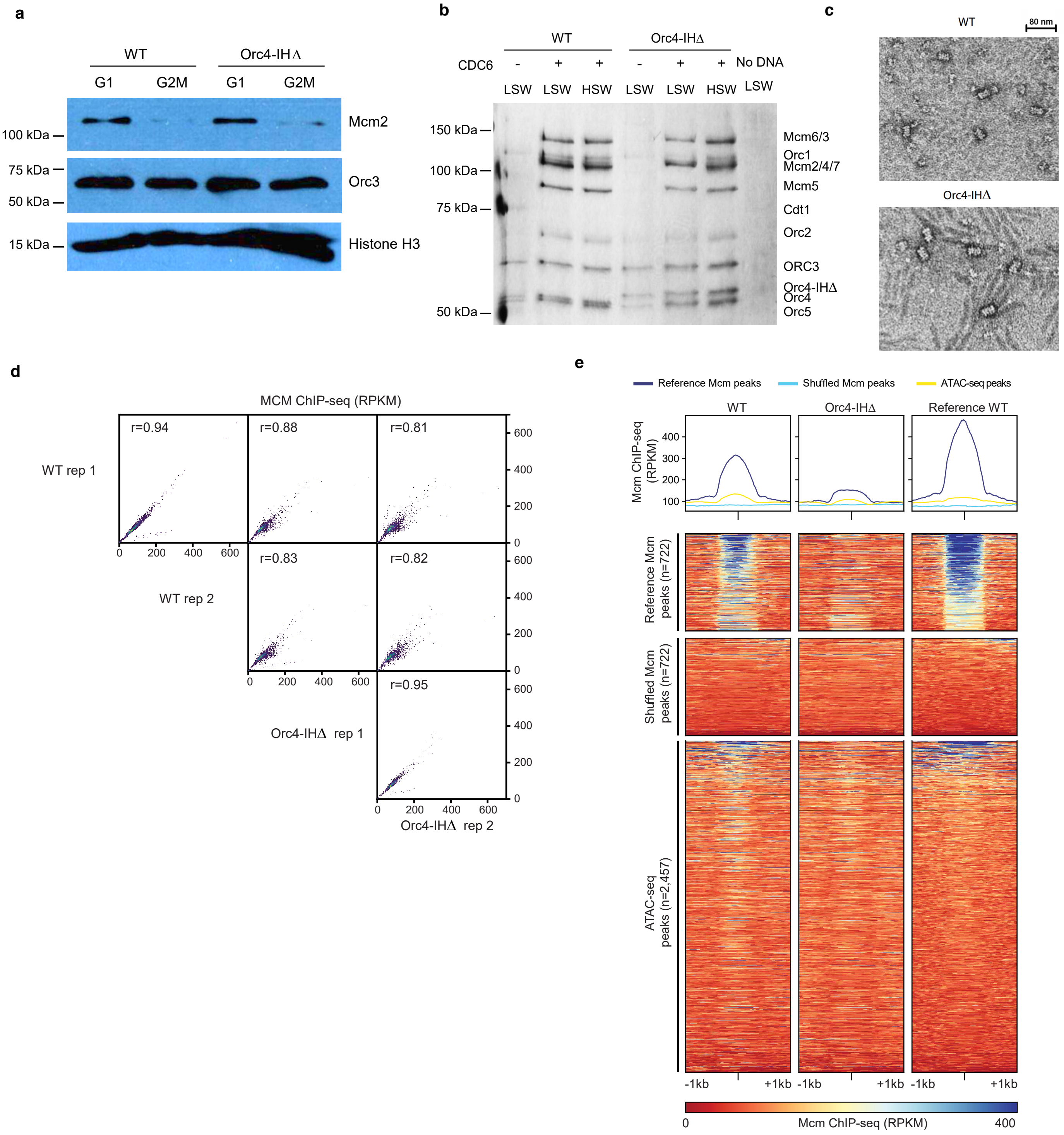
a
WT
Orc4-IHΔ
G1
G2M
G1
G2M
100 kDa
75 kDa
50 kDa
15 kDa
Mcm2
Orc3
Histone H3
b
WT
Orc4-IHΔ
CDC6
-
+
+
-
+
+
No DNA
LSW
LSW
HSW
LSW
LSW
HSW
LSW
150 kDa
100 kDa
75 kDa
50 kDa
Mcm6/3
Orc1
Mcm2/4/7
Mcm5
Cdt1
Orc2
ORC3
Orc4-IHΔ
Orc4
Orc5
c
WT
80 nm
Orc4-IHΔ
d
MCM ChIP-seq (RPKM)
WT rep 1
r=0.94
r=0.88
r=0.81
WT rep 2
r=0.83
r=0.82
Orc4-IHΔ rep 1
r=0.95
Orc4-IHΔ rep 2
0
200
400
600
e
Reference Mcm peaks
Shuffled Mcm peaks
ATAC-seq peaks
WT
Orc4-IHΔ
Reference WT
Mcm ChIP-seq (RPKM)
100
200
300
400
Reference Mcm peaks (n=722)
Shuffled Mcm peaks (n=722)
ATAC-seq peaks (n=2,457)
-1kb
+1kb
0
Mcm ChIP-seq (RPKM)
400

Supplementary Fig 2. In vivo and in vitro MCM loading promoted by Orc4-IHΔ.

a. Chromatin association of MCM and ORC in G1 and G2M phase of WT and mutant cells. Chromatin fractions of the indicated cells were analysed by SDS–PAGE and immunoblotting of Mcm2 and Orc3. Histone H3 served as loading control. Blots shown are representative of n=2 biological replicates. Source data are provided as source data file.

b. In vitro pre-RC assembly assays were performed in reactions containing Cdc6 and Cdt1-Mcm2-7 with either ORC or ORC-IHΔ. LSW (low salt wash) and HSW (high salt wash) were performed to remove unbound proteins. DNA bound proteins were analysed by silver staining and labelled as indicated. Blots shown are representative of n=2 biological replicates. Source data are provided as source data file.

c. The assembled MCM double hexamers were visualized by negative staining EM. Micrographs shown are representative of n=2 biological replicates.

d. Scatter plots show pairwise comparisons of averaged MCM ChIP-seq normalized signal (RPKM) between each replicate of WT and Orc4-IHΔ. The genome is segmented into 5kb bins for analysis. Pearson correlation coefficient for each pair is shown.

e. Aggregation plots and heatmaps show the MCM enrichment patterns in WT and Orc4-IHΔ. We integrated a published WT MCM ChIP-seq dataset[1] to validate the quality of our MCM ChIP-seq. At MCM peaks defined from this reference dataset (top), we observed similar MCM ChIP-seq signals (RPKM) in WT but dramatically reduced binding in Orc4-IHΔ. Number- and size-matched shuffled genome loci (middle) were included as controls. In addition, MCM enrichment patterns at ATAC-seq peaks were included to indicate enrichment of MCM at open chromatin. Line plots aggregate the signal of each cluster, while heatmaps show the signal at each locus in individual row.

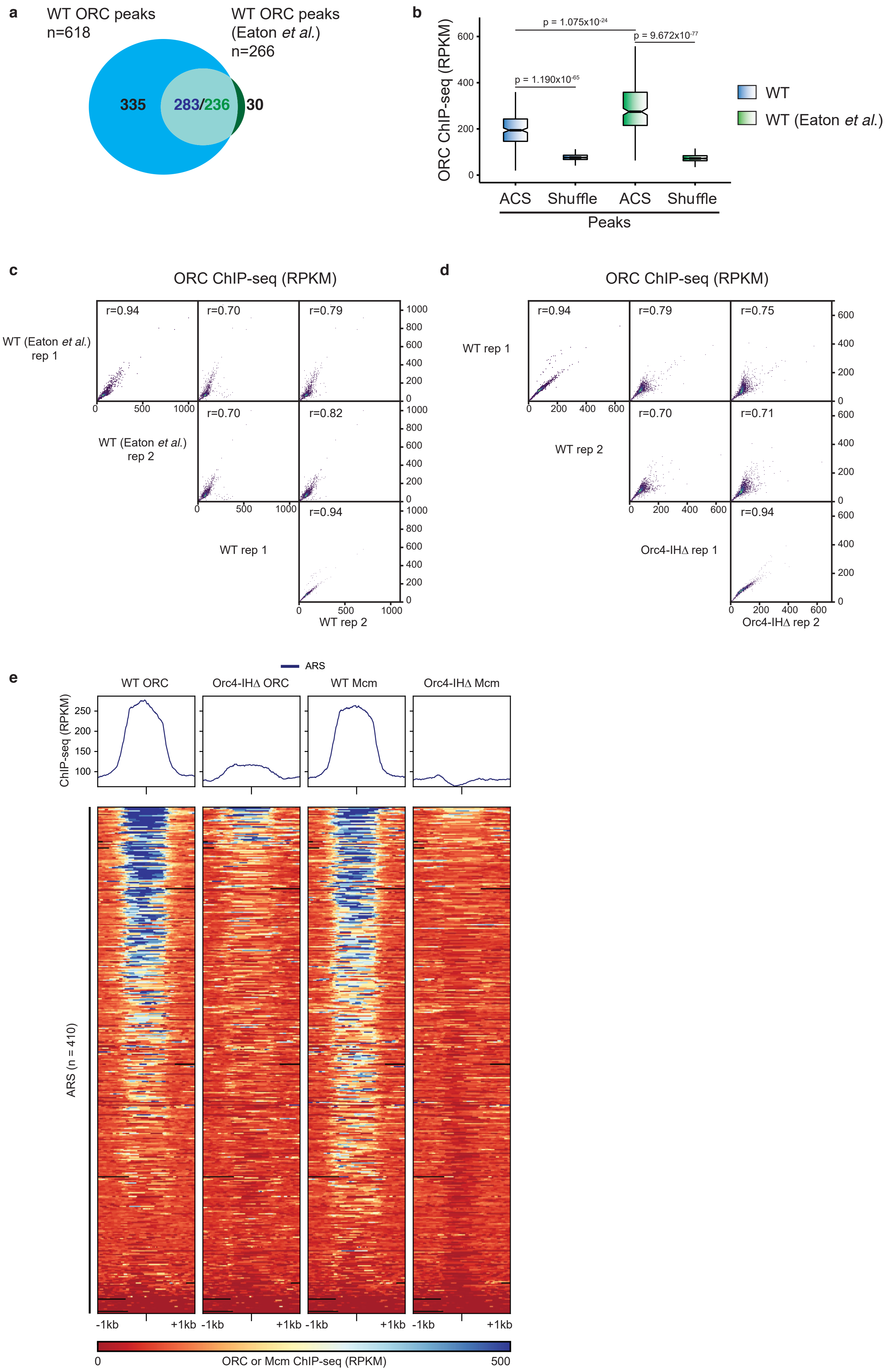
a
WT ORC peaks
n=618
WT ORC peaks
(Eaton et al.)
n=266
335
283/236
30
b
ORC ChIP-seq (RPKM)
p = 1.075x10-24
p = 9.672x10-77
p = 1.190x10-65
600
400
200
0
ACS
Shuffle
ACS
Shuffle
Peaks
WT
WT (Eaton et al.)
c
ORC ChIP-seq (RPKM)
WT (Eaton et al.) rep 1
WT (Eaton et al.) rep 2
WT rep 1
WT rep 2
r=0.94
r=0.70
r=0.79
r=0.70
r=0.82
r=0.94
0
500
1000
200
400
600
800
d
ORC ChIP-seq (RPKM)
WT rep 1
WT rep 2
Orc4-IHΔ rep 1
Orc4-IHΔ rep 2
r=0.94
r=0.79
r=0.75
r=0.70
r=0.71
r=0.94
0
200
400
600
e
ARS
WT ORC
Orc4-IHΔ ORC
WT Mcm
Orc4-IHΔ Mcm
ChIP-seq (RPKM)
250
200
150
100
ARS (n = 410)
-1kb
+1kb
0
ORC or Mcm ChIP-seq (RPKM)
500

Supplementary Fig 3. Comparison of ORC ChIP-seq datasets.

a. Venn diagram showing the high degree of overlap between ORC ChIP-seq peaks defined in WT from this study (n=618) (blue) and from a previously published dataset (n=266) (green)[2]. Of these, 283 and 236 peaks overlapped by at least 1 bp between our dataset and the reference dataset, respectively (cyan). Overlapping peaks within a region may or may not align.

b. Boxplot showing ORC ChIP-seq enrichment (RPKM) at ACS containing regions (n=249) and shuffled genomic loci (n=249) of WT from this study (blue) and from a previously published dataset (green). Pairwise quantitative comparisons show consistent trends of significantly higher ORC binding at ACS-containing regions than shuffled controls in both datasets. P-values were calculated by two-sided Wilcoxon rank sum test.The upper and the lower bound of the box denotes the 75th and 25th percentiles of the data, respectively. The black lines in the box denotes the median.

c. Scatter plots show pairwise comparisons of averaged ORC ChIP-seq normalized signals (RPKM) between each replicate of WT from this study and WT from a previously published study[2]. The genome is segmented into 5kb bins for analysis. Pearson correlation coefficient for each pair is shown.

d. Scatter plots show pairwise comparisons of averaged ORC ChIP-seq normalized signal (RPKM) between each replicate of WT and Orc4-IHΔ. The genome is segmented into 5kb bins for analysis. Pearson correlation coefficient for each pair is shown.

e. Aggregation plots and heatmaps show the ORC and MCM enrichment patterns in WT and Orc4-IHΔ. At OriDB annotated ARS (±1kb), we detected about half of these loci with concordantly high ORC and MCM ChIP-seq signal (RPKM) in WT. However, little to no ORC and MCM binding was found in the Orc4-IHΔ. Line plots aggregate the signal of all ARSs, while heatmaps show the signal at each element in individual rows.

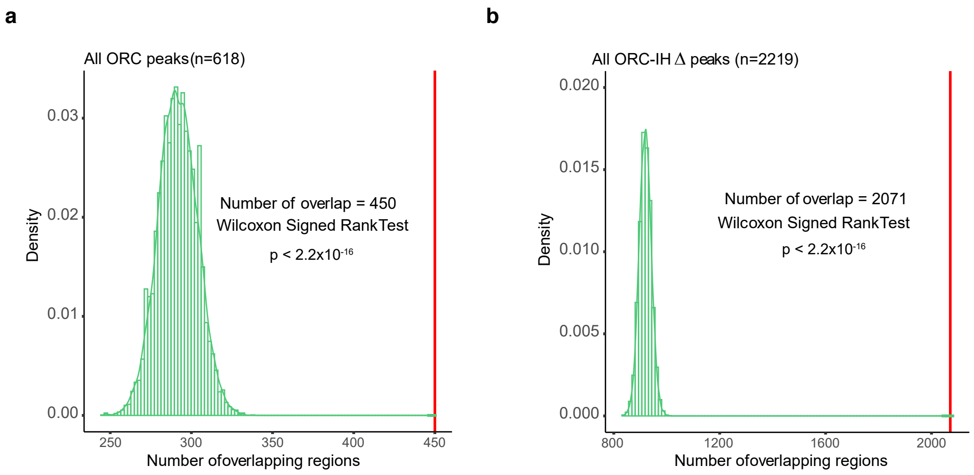

Supplementary Fig 4. Enrichment of ORC and ORC-IHΔ binding sites at intergenic regions.

Overlaps between intergenic regions (sacCer2) and a) all ORC peaks (n=618) and b) all Orc4-IHΔ peaks (n=2,219). Histograms/density curves illustrate the distribution (counts) of number-matched, randomly sampled regions, which were selected 10,000 times. This distribution represents the probability of finding a given number of peaks in intergenic regions by chance. The red line indicates the actual number of peaks that overlap with intergenic definitions. P-value were calculated by two-sided Wilcoxon Signed Rank Test.

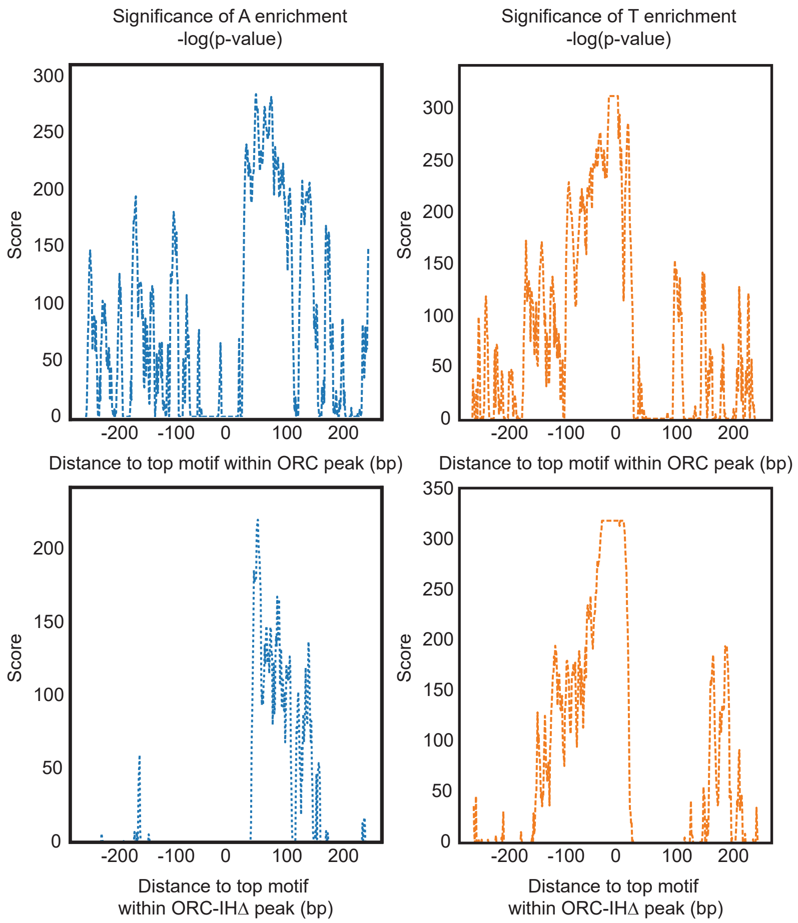

Supplementary Fig 5. WT and mutant ORC binding motifs show statistically significant enrichment of polyA/T.

The statistical significance of the A/T enrichment at top motifs within WT and Orc4-IHΔ ORC ChIP-seq peaks (±250bp). Position 0 denotes the first base of the particular motif, which corresponds to the positions shown in Figure 4a. To calculate the significance of A/T enrichment at each position, number- and size- matched shuffle genomic loci were sampled from the reference genome 300 times. For each position, the distribution of base composition of these randomly shuffled regions were used to perform one-tailed Student's t-test against the base composition generated from the top motif within WT and Orc4-IHΔ ORC ChIP-seq peaks. The scores were calculated by -log(p-value). p-values have been adjusted by Benjamini-Hochberg method.

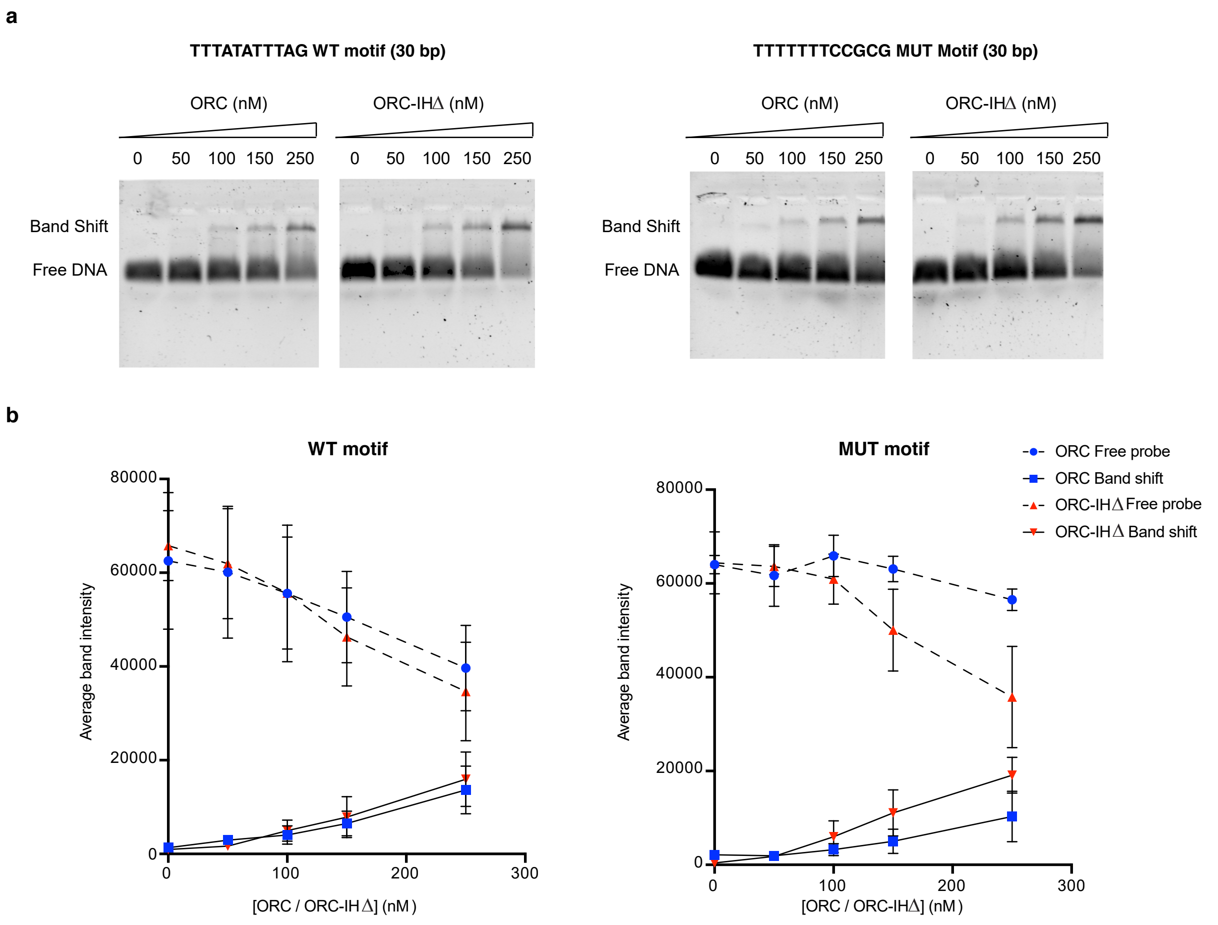

Supplementary Fig 6. WT and mutant ORC have differential binding affinities to ACS and polyT tract substrates.

a. EMSA analysis of WT or mutant ORC using 30 bp dsDNA substrates containing ACS (TTTATATTTAG) or polyT tract (TTTTTTTCCGCG) derived from ORC ChIP-seq analyses. Protein concentrations are indicated. Source data are provided as source data file.

b. Line plots with data points correspond to mean value of Sybr Safe signal intensity of free probe and band shift ± SD from n=3 replicates. Source data are provided as source data file.

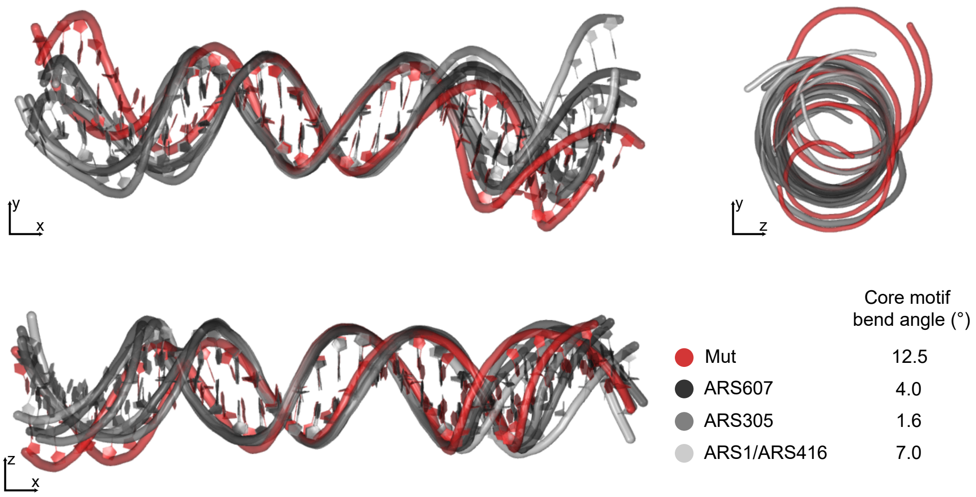

Supplementary Fig 7. Three different views of rebuilt DNA structures and their bend angles for core motif sites.

Structures are aligned based on their core motif sites (10 bp in the center). The x- and y-axes were generated based on the highest and second-highest principal components of all atoms in the aligned structures. Bend angles are calculated using Curves 5.3 for their core motif sites. Bend angles represent the angles between two vectors formed between two bp at each end of the core motif (bp 1 and 2, and bp n-1 and n, respectively) of the core motif sites, where the vector is defined between a reference point from one bp and the equivalent reference point on another bp. For definition of nucleic acid reference system, see Lavery et al. (1989)[3].

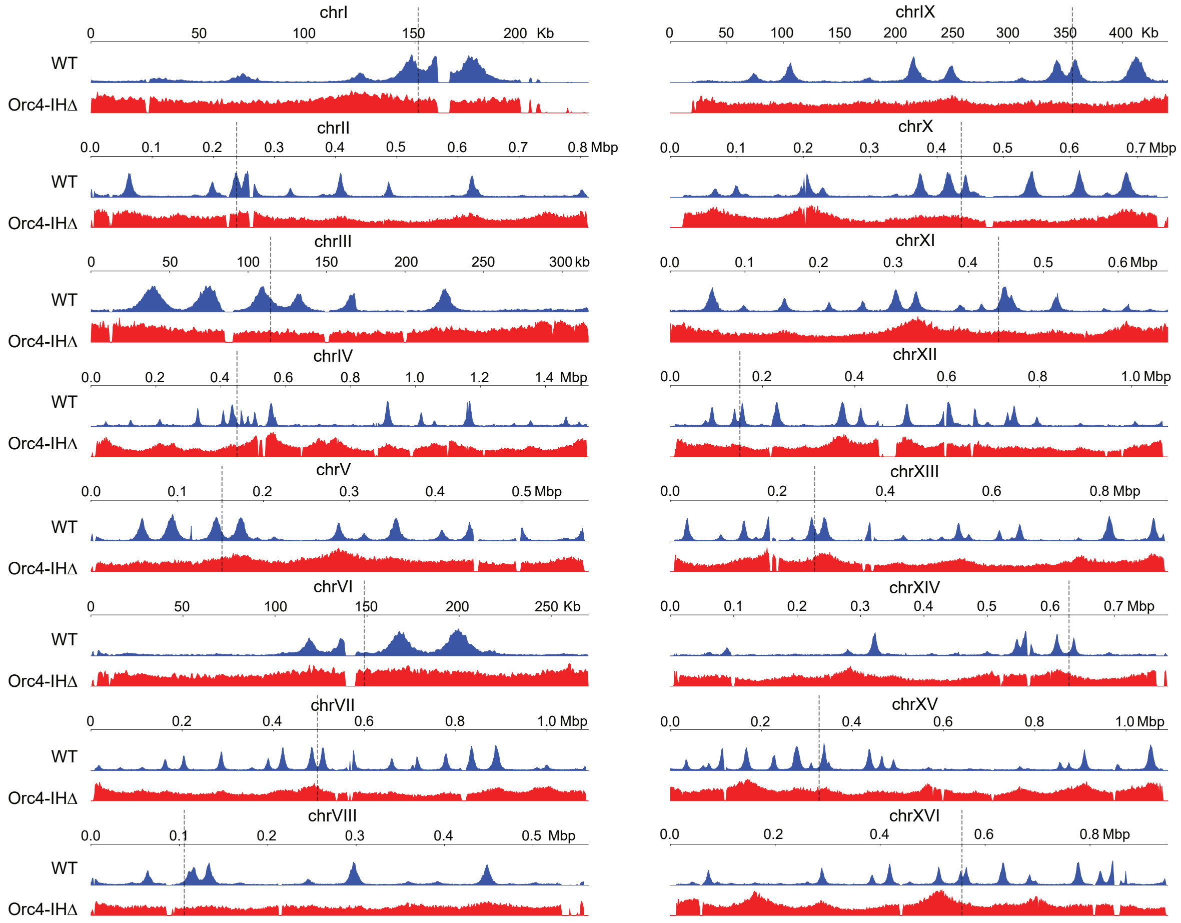

Supplementary Fig 8. Orc4-IHΔ shows global perturbation of BrdU-seq signal.

Chromosome-wide genome browser screenshots show the normalized BrdU-seq signal for WT (blue) and Orc4-IHΔ (red) for all chromosomes. Centromeres are denoted with a dotted line. While punctate peaks were observed in WT, dramatically altered patterns were seen in the mutant. Y-axis for all tracks were adjusted to the same scale (0-1).

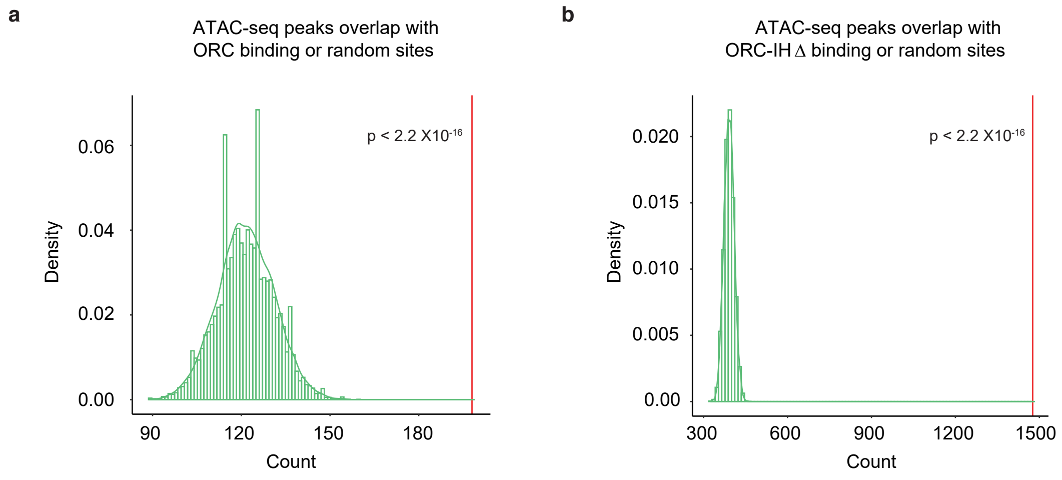

Supplementary Fig 9. WT and mutant ORC binding is enriched at sites of open chromatin.

Overlaps between ATAC-sea peaks and all ORC peaks (n=618) or all Orc4-IHΔ peaks (n=2,219). Histograms/density curves illustrate the distribution (counts) of number-matched, randomly sampled regions, which were selected 10,000 times. This distribution represents the probability of finding a given number of ORC peaks that overlap with ATAC-seq peaks by chance. The red line indicates the actual number of ATAC-seq peaks that overlap with ORC ChIP-seq peaks defined in a) WT (n=198) and b) Orc4-IHΔ (n=1469). P-value were calculated by two-sided Wilcoxon Signed Rank Test.

Yeast Strain	Genotype
W303-1a	*MATa ade2-1 ura3-1 his3-11, 15 trp1-1 leu2-3, 112 can1-100*
GALS-ORC4	W303-1a *GALS-ORC4-KanMX4*
GALS-ORC1	W303-1a *GALS-ORC1-KanMX4*
CL_WT_O6_MYC	W303-1a *GALS-ORC4-KanMX4 ura3-1::ORC4-URA3 ORC6-MYC-hphNT1*
CL_MUT_O6_MYC	W303-1a *GALS-ORC4-KanMX4 ura3-1::orc4-IH∆-URA3 ORC6-MYC-hphNT1*
CL_WT_M7_FLAG	W303-1a *GALS-ORC4-KanMX4 ura3-1::ORC4-URA3 MCM7-FLAG-hphNT1*
CL_MUT_M7_FLAG	W303-1a *GALS-ORC4-KanMX4 ura3-1::orc4-IH∆-URA3 MCM7-FLAG-hphNT1*
ySD-ORC	W303-1a *bar::Hyg pep4::KanMX TRP1 Gal1-10 ORC5, ORC6 HIS3 Gal1-10 ORC3, ORC4 URA3 Gal1-10 CBP-ORC1, ORC2*
ySD-ORC-MutORC4	ySD-ORC *LEU2 Gal1-10 FLAG-orc4-IH∆*
CL_GALS_ORC1_WT	W303-1a *GALS-ORC1-KanMX4 ura3-1::ORC1-URA3*
CL_GALS_ORC1_MUT	W303-1a *GALS-ORC1-KanMX4 ura3-1::orc1-BP4∆-URA3*
BrdU_WT	CL_WT_O6_MYC *trp1-1::BrdU-Inc-TRP1*
BrdU_MUT	CL_MUT_O6_MYC *trp1-1::BrdU-Inc-TRP1*

Plasmid	Description
pYM-N30	Cassette for endogenous *ORC1* and *ORC4* promoter modification.
pRS406	Plasmid for integration in *GALS-ORC4* strain.
pRS406-ORC4	Plasmid for integration and expression of WT Orc4 in *GALS-ORC4* strain.
pRS406-orc4-IH∆	Plasmid for integration and expression of mutant Orc4 in *GALS-ORC4* strain.
pYM20	Cassette for C terminal fusion of MYC tag to Orc6 in *GALS-ORC4* strain.
pTF272	Cassette for C terminal fusion of FLAG tag to Mcm7 in *GALS-ORC4* strain.
pRS405-GAL-Sc-orc4-IH∆	Plasmid for integration in ySD-ORC strain.
pRS404-BrdU-Inc	Plasmid for integration of BrdU incorporation cassette.

Supplementary Table 1. Yeast strains and plasmids used in this study.

Primer Name	Forward primer (5' to 3')	Reverse primer (5' to 3')	Description
Orc1_GALS_tag	TCATTCCCTATTGATTAATACTCATTACAAAAACCACAATAGAGTAGATAAGATGCGTACGCTGCAGGTCGAC	CCTGCTCATCAGTTGTTATTATCTCCCAACCCTGTAAATCCTTCAACGTTTTTGCCATCGATGAATTCTCTGTCG	Primers for N-terminal replacement of endogenous *ORC1* promoter to *GALS* promoter
Orc4_GALS_tag	TAGAAATATCAAATATAAACTACTAATATCGGTAATATTCAAAAGAAGAAGCATGCGTACGCTGCAGGTCGAC	GCCTCTTTATTGGGAGAAGATTGACTTGCGGTGATAGACGAGCTTCGCTTATAGTCATCGATGAATTCTCTGTCG	Primers for N-terminal replacement of endogenous *ORC4* promoter to *GALS* promoter
Orc6_MYC_tag	TGGAAGAAAAGAATTGAAATGGATTTGGCATTAACAGAACCTTTACGTACGCTGCAGGTCGAC	AAGATATGTCAGGTATTGGTCAAATATATACTTTTAGTTAATACTGGATATGTTAATCGATGAATTCGAGCTCG	Primers for C-terminal tagging of endogenous Orc6 with MYC tag
Mcm7_FLAG_tag	CTAATGTGAGCGCCCAAGATTCTGATATCGATCTACAAGACGCTCGTACGCTGCAGGTCG	AGTTACAGAAGGTAAATTTGAATGTGCAGCTATATGGAAATGATCGATGAATTCGAGCTC	Primers for C-terminal tagging of endogenous Mcm7 with FLAG tag
Orc4_FL	AAATTAGAGCTTCAATTTAATTATATCAGTTATTACAAAACTGCTCAATTGACTGTTG	CCTTACGCATCTGTGCGGTATTTCACACCGCATAGGTCACTCATATTATTTTACTG	Primers for cloning of full-length *ORC4* into pRS406
Orc4_del_IH	GAGAAATCAGCCGTTGGTTTGAGACCGTTTGACTTGA	TCAAGTCAAACGGTCTCAAACCAACGGCTGATTTCTC	Primers for cloning of *orc4-IH*Δ into pRS406
ARS_1	GGTGAAATGGTAAAAGTCAACCCCCTGCG	GCTGGTGGACTGACGCCAGAAAATGTT	ChIP primers for ARS1 positive loci
Con_1	CATCAATTGTGCACTCGGAC	GAACACGGCAATTGTAGGTGG	ChIP primers for negative loci
ARS_607	CAGAGAAACTATTACATTGTACATTCTCC	TGTGCCGAATAATGTGTAAGTCTC	ChIP primers for ARS607 positive loci
Con_607	TGGTCAACCTCAACTTTCACA	GATTTCTTGCGCATGACTCA	ChIP primers for negative loci

Supplementary Table 2. Primers used in this study.

Bioinformatic Analysis

ChIP-seq and BrdU-IP-seq data processing
Raw sequencing reads were mapped against yeast reference genome sacCer2 (UCSC) using Bowtie (version 1.2.2) (parameters "--phred33-quals -v 3 -m 1 -p 24 --best --strata"). PCR duplicates were marked and removed using Picard tools MarkDuplicates (version 2.9.0-1-gf5b9f50-SNAPSHOT). Only uniquely mapped reads were kept. Unless specifically stated, raw sequencing reads from different replicates (If any) of the same sample were merged and mapped as described above. Mapped reads were visualized using samtools (version 1.3.1) and bedtools (version 2.27.1). RPKM for every merged sample is calculated per 100bp non-overlapping window across the genome. Deeptools (version 3.0.1) was used to generate heatmaps and profiles[4-9].
For BrdU-IP-seq tracks, the mapped reads were binned into 100bp non overlapping bins. Each bin from the IP library was divided to its corresponding input bin, yielding an IP:Input ration for each bin. The bins were median smoothed over a 500bp window. In each library, the bin with the highest value was assigned a value of 1 and the remaining bins are scaled to that bin by dividing with the highest value[10].

Peak calling and ACS definition
For WT and Orc4-IHΔ ChIP-seq datasets, peaks were defined with each replicate using MACS2 (version 2.1.1.20160309) (parameters "--nomodel --extsize 147 -q 0.005 -g 12.07e6"). Only those peaks that were called in both replicates of a sample and did not overlap with tRNA genes (GtRNAdb) were kept for downstream analysis. For ATAC-seq peak calling, MACS2 (version 2.1.1.20160309) was used wtiht the following parameters "-g 12100000 --keep-dup all -B --SPMR --nomodel --shift -75 --extsize 150 -p 0.01". Reference ACS locations were obtained from GSM424494[2]. Genome coordinates were converted to sacCer2 form sacCer1 using LiftOver (UCSC). ACSs locate 1,000 bp or less near chromosome ends were filtered. Deeptools (version 3.0.1) were used to generate heatmaps and profiles[11].

For reference publically available datasets, the same manner of processing were applied as our own data. Peaks were defined with each replicate using MACS2 (version 2.1.1.20160309) (parameters "--nomodel --extsize 147 -q 0.0005 -g 12.07e6").

Number-matched shuffled loci refer to a set of randomly picked regions in the genome that have the same number of events and share the same length as the original set. For instance, for the 618 WT Orc peaks, we randomly picked 618 regions by shuffling the genomic coordinates of the of peaks, whilst keeping the peak size constant. The result would be the same number of peaks that are scattered throughout the genome but share the same lengths as the test set. We would subsequently use this number-matched and size-matched shuffled set as a representative of the genome background.

RNA-seq data processing and integration
A public RNA-seq data is used to determine transcriptional strength in WT yeast. SRR363968 is used because the data was generated using the same strain W303-1A as in our study. The data obtained is mapped against sacCer2 using STAR aligner (version 2.7.5a). The gene transcript annotation was obtained from Ensembl Genomes. Gene level expressions, in transcript per million (TPM), were quantified using RSEM (version 1.3.3). Heatmaps of WT and Orc4-IHΔ Orc ChIP-seq signal were plotted, where each row represents one transcript. These rows are ranked and sorted by TPM in descending order. From the Orc4-IHΔ heatmap, a positive association between transcriptional strength and Orc4-IHΔ binding was discovered.

De novo motif search

Homer (version 4.9.1-5) findMotifsGenome.pl was used to search for motifs enriched in WT and Orc4-IHΔ ChIP-seq peaks. (parameters "-size 200")[12]

ATAC-seq and MNase-seq data processing

Raw sequencing reads were mapped against yeast reference genome sacCer2 (UCSC) using Bowtie2 (version 2.3.4.1) (parameters " -t -q -p 24 -N 1 -L 25 -X 2000 --no-discordant --no-mixed --un-conc"). PCR duplicates were marked and removed using Picard tools MarkDuplicates (version 2.9.0-1-gf5b9f50-SNAPSHOT). Reads mapped to chrM were removed[13].
For MNase-seq, RPKM of 100bp non-overlapping windowed genome is calculated.

Nucleosome positioning index

Similar to the nucleosome positioning analysis done previously by Ishii et al., fragments between 141bp and 190bp were extracted from the mapped reads, the middle 60 base pair of each fragment was assigned a value of 1. For each position, the sum of values is normalized by dividing by the genomic average. Genomic average was calculated as the total number of reads with fragment sizes between 141bp and 190bp, multiplied by 60 and divided by the yeast reference genome size[14].

Base composition analysis

The top motif identified in each peak set was used in the subsequent analysis. The motif used for WT is TTTATGTTTAGK, while the motif used for Orc4-IHΔ is TTTTTYSS. The presence and location of each motif was identified in the reference genome. The list of locations was then compared to Orc ChIP-seq peaks. Locations that do not overlap with any peak or were too close to the end of chromosome were filtered out. For the remaining locations, using the first base of motif as position 0, we analyzed 250bp upstream and downstream. The base composition of each base was calculated for each position and the curves, representing each base, were smoothed using rolling-average of 9 bases window with step-size of 1 base.

To calculate the significance of A/T enrichment at each position, number- and size-matched shuffle genomic loci were sampled from the reference genome 300 times. For each position, the distribution of base composition of these random shuffled regions were used to perform one-tailed Student's t-test against the base composition generated from the top motif within WT and Orc4-IHΔ ORC ChIP-seq peaks. The scores were calculated by –log(p-value).

Supplementary References

1 Belsky, J. A., MacAlpine, H. K., Lubelsky, Y., Hartemink, A. J. & MacAlpine, D. M. Genome-wide chromatin footprinting reveals changes in replication origin architecture induced by pre-RC assembly. *Genes Dev* **29**, 212-224, doi:10.1101/gad.247924.114 (2015).

2 Eaton, M. L., Galani, K., Kang, S., Bell, S. P. & MacAlpine, D. M. Conserved nucleosome positioning defines replication origins. *Genes Dev* **24**, 748-753, doi:10.1101/gad.1913210 (2010).

3 Lavery, R. & Sklenar, H. Defining the structure of irregular nucleic acids: conventions and principles. *J Biomol Struct Dyn* **6**, 655-667, doi:10.1080/07391102.1989.10507728 (1989).

4 Kent, W. J., Zweig, A. S., Barber, G., Hinrichs, A. S. & Karolchik, D. BigWig and BigBed: enabling browsing of large distributed datasets. *Bioinformatics* **26**, 2204-2207, doi:10.1093/bioinformatics/btq351 (2010).

5 Kuhn, R. M., Haussler, D. & Kent, W. J. The UCSC genome browser and associated tools. *Brief Bioinform* **14**, 144-161, doi:10.1093/bib/bbs038 (2013).

6 Langmead, B., Trapnell, C., Pop, M. & Salzberg, S. L. Ultrafast and memory-efficient alignment of short DNA sequences to the human genome. *Genome Biol* **10**, R25, doi:10.1186/gb-2009-10-3-r25 (2009).

7 Li, H. *et al.* The Sequence Alignment/Map format and SAMtools. *Bioinformatics* **25**, 2078-2079, doi:10.1093/bioinformatics/btp352 (2009).

8 Quinlan, A. R. & Hall, I. M. BEDTools: a flexible suite of utilities for comparing genomic features. *Bioinformatics* **26**, 841-842, doi:10.1093/bioinformatics/btq033 (2010).

9 Ramirez, F. *et al.* deepTools2: a next generation web server for deep-sequencing data analysis. *Nucleic Acids Res* **44**, W160-165, doi:10.1093/nar/gkw257 (2016).

10 Peace, J. M., Villwock, S. K., Zeytounian, J. L., Gan, Y. & Aparicio, O. M. Quantitative BrdU immunoprecipitation method demonstrates that Fkh1 and Fkh2 are rate-limiting activators of replication origins that reprogram replication timing in G1 phase. *Genome Res* **26**, 365-375, doi:10.1101/gr.196857.115 (2016).

11 Zhang, Y. *et al.* Model-based analysis of ChIP-Seq (MACS). *Genome Biol* **9**, R137, doi:10.1186/gb-2008-9-9-r137 (2008).

12 Heinz, S. *et al.* Simple combinations of lineage-determining transcription factors prime cis-regulatory elements required for macrophage and B cell identities. *Mol Cell* **38**, 576-589, doi:10.1016/j.molcel.2010.05.004 (2010).

13 Langmead, B. & Salzberg, S. L. Fast gapped-read alignment with Bowtie 2. *Nat Methods* **9**, 357-359, doi:10.1038/nmeth.1923 (2012).

14 Ishii, H., Kadonaga, J. T. & Ren, B. MPE-seq, a new method for the genome-wide analysis of chromatin structure. *Proc Natl Acad Sci U S A* **112**, E3457-3465, doi:10.1073/pnas.1424804112 (2015).
