# Supplementary material for: Humanizing the yeast origin recognition complex
Source: Nat Commun. 2021 Jan 4;12:33. doi: 10.1038/s41467-020-20277-y (PMC7782691; doi:10.1038/s41467-020-20277-y)
Supplement: Supplementary file 3 — Reporting Summary [file 41467_2020_20277_MOESM3_ESM.pdf]

nature research

Corresponding author(s): Bik-Kwoon Tye
Danny Leung
Yuanliang Zhai

Last updated by author(s): Oct 20, 2020

Reporting Summary

Nature Research wishes to improve the reproducibility of the work that we publish. This form provides structure for consistency and transparency in reporting. For further information on Nature Research policies, see our Editorial Policies and the Editorial Policy Checklist.

Statistics

For all statistical analyses, confirm that the following items are present in the figure legend, table legend, main text, or Methods section.

n/a	Confirmed	
☐	☒	The exact sample size (*n*) for each experimental group/condition, given as a discrete number and unit of measurement
☐	☒	A statement on whether measurements were taken from distinct samples or whether the same sample was measured repeatedly
☐	☒	The statistical test(s) used AND whether they are one- or two-sided<br>*Only common tests should be described solely by name; describe more complex techniques in the Methods section.*
☐	☒	A description of all covariates tested
☐	☒	A description of any assumptions or corrections, such as tests of normality and adjustment for multiple comparisons
☐	☒	A full description of the statistical parameters including central tendency (e.g. means) or other basic estimates (e.g. regression coefficient) AND variation (e.g. standard deviation) or associated estimates of uncertainty (e.g. confidence intervals)
☐	☒	For null hypothesis testing, the test statistic (e.g. *F*, *t*, *r*) with confidence intervals, effect sizes, degrees of freedom and *P* value noted<br>*Give P values as exact values whenever suitable.*
☐	☒	For Bayesian analysis, information on the choice of priors and Markov chain Monte Carlo settings
☐	☒	For hierarchical and complex designs, identification of the appropriate level for tests and full reporting of outcomes
☐	☒	Estimates of effect sizes (e.g. Cohen's *d*, Pearson's *r*), indicating how they were calculated

Our web collection on statistics for biologists contains articles on many of the points above.

Software and code

Policy information about availability of computer code

Data collection	CELLQuest (V3.3) was used for collecting flow cytometry data
Data analysis	Bowtie (version 1.2.2), Picard tools MarkDuplicates (version 2.9.0-1-gf5b9f50-SNAPSHOT), samtools (version 1.3.1), bedtools (version 2.27.1), Deeptools (version 3.0.1), MACS2 (version 2.1.1.20160309), Homer (version 4.9.1-5), Bowtie2 (version 2.3.4.1), Flowjo (v10.0.7r2),STAR aligner (version 2.7.5a), RSEM (version 1.3.3)

For manuscripts utilizing custom algorithms or software that are central to the research but not yet described in published literature, software must be made available to editors and reviewers. We strongly encourage code deposition in a community repository (e.g. GitHub). See the Nature Research guidelines for submitting code & software for further information.

Data

Policy information about availability of data

All manuscripts must include a data availability statement. This statement should provide the following information, where applicable:

- Accession codes, unique identifiers, or web links for publicly available datasets
- A list of figures that have associated raw data
- A description of any restrictions on data availability

All sequencing data generated in this study were deposited to GEO under the accession number GSE149163
Previously published datasets of WT yeast RNA-seq, WT ORC-ChIP-seq and WT MCM2-7 ChIP-seq were obtained from SRR363968 , GSE16926 and GSE38032, respectively.

Field-specific reporting

Please select the one below that is the best fit for your research. If you are not sure, read the appropriate sections before making your selection.

☒ Life sciences ☐ Behavioural & social sciences ☐ Ecological, evolutionary & environmental sciences

For a reference copy of the document with all sections, see nature.com/documents/nr-reporting-summary-flat.pdf

Life sciences study design

All studies must disclose on these points even when the disclosure is negative.

Sample size	Biochemical experiments, including yeast cell culturing, protein purification, chromatin binding assay, and SDS-PAGE were done in biological replicates (N>3)
Data exclusions	No data was excluded from the analysis.
Replication	all attempts of replication was succesful.
Randomization	Randomization was not necessary for genomics studies of this type. As the samples are divided by their genetic backgrounds, they cannot be randomized.
Blinding	Investigators were not blinded to the group allocations as they samples are divided by their genetic backgrounds.

Reporting for specific materials, systems and methods

We require information from authors about some types of materials, experimental systems and methods used in many studies. Here, indicate whether each material, system or method listed is relevant to your study. If you are not sure if a list item applies to your research, read the appropriate section before selecting a response.

Materials & experimental systems

n/a	Involved in the study	
☐	☒	Antibodies
☒	☐	Eukaryotic cell lines
☒	☐	Palaeontology and archaeology
☒	☐	Animals and other organisms
☒	☐	Human research participants
☒	☐	Clinical data
☒	☐	Dual use research of concern

Methods

n/a	Involved in the study	
☐	☒	ChIP-seq
☐	☒	Flow cytometry
☒	☐	MRI-based neuroimaging

Antibodies

Antibodies used	Anti-c-myc Mouse monoclonal antibody (clone 9E10) (Cat#11667149001, Lot#37569500), Roche;<br>Monoclonal ANTI-FLAG® M2 antibody produced in mouse, clone M2, (F3165) Sigma-Aldrich;<br>Anti-yeast Mcm2 and anti-yeast Orc3 monoclonal antibodies (gifts from Bruce Stillman);<br>Anti-yeast Rad53 antibody (yC-19, Lot#E0410), Santa Cruz;<br>Anti-Histone H3 monoclonal antibody (ab1791), Abcam;<br>Anti-BrdU Monoclonal Antibody (ZBU30) (Cat# 03-3900, Lot#Q1215270), Invitrogen.
Validation	Western blot was done to validate specificity of antibodies towards Myc, FLAG, Mcm2, Orc3, H3 and Rad53.

ChIP-seq

Data deposition

☒ Confirm that both raw and final processed data have been deposited in a public database such as GEO.

☒ Confirm that you have deposited or provided access to graph files (e.g. BED files) for the called peaks.

Data access links<br>*May remain private before publication.*	https://www.ncbi.nlm.nih.gov/geo/query/acc.cgi?acc=GSE149163
Files in database submission	processed data for ORC ChIP-seq:<br>GSM4491309_merged_ChIP_Orc-WT.normarpkm.bw

GSM4491313_merged_ChIP_Orc-Mut.normarpkm.bw
GSM4491309_Orc-WT.common.notRNA.bed.gz
GSM4491313_Orc-Mut.common.notRNA.bed.gz
processed data for MCM ChIP-seq:
GSM4491310_merged_ChIP_Mcm-WT.normarpkm.bw
GSM4491314_merged_ChIP_Mcm-Mut.normarpkm.bw
processed data for BrdU-IP-seq:
GSM4491311_ChIP_Brdu_WT_rep1.normarpkm.bw
GSM4491315_ChIP_Brdu_Mut_rep1.normarpkm.bw
processed data for MNase-seq:
GSM4491312_Mnase-WT-G1.60bp.bw
GSM4491316_Mnase-Mut-G1.60bp.bw
processed data for ATAC-seq:
GSM4491317_20191205-ATACSEQ2019006-Mut-G2M.PEuniq.noM.nodup.Cuts.bed.MACS2_p0.01_S-75E150._peaks.narrowPeak.gz
GSM4491318_20191205-ATACSEQ2019006-WTG2M.PEuniq.noM.nodup.Cuts.bed.MACS2_p0.01_S-75E150._peaks.narrowPeak.gz

Genome browser session (e.g. UCSC)

A genome browser session containing files deposited in GEO
https://genome.ucsc.edu/s/mfcheungeric/GSE149163_revised

Methodology

Replicates

2 replicates for Myc-tag ORC ChIP-seq for G2/M arrested WT and mutant yeast cells. 2 replicates for FLAG-tag MCM ChIP-seq for G1 arrested WT and mutant yeast. 1 replicate for BrdU IP-seq for early S arrested WT and mutant yeast cells. 1 replicate for MNase-seq for G1 arrested WT and mutant yeast cells. 1 replicate for ATAC-seq for G2M arrested WT and mutant yeast cells.

Sequencing depth

ATAC-Mut-G2M, total read: 5506595,uniquely mapped: 3500154, read length: 75bp, layout:Pair-end;
ATAC-WT-G2M, total read: 6120172,uniquely mapped: 3378616, read length: 75bp, layout:Pair-end;
Brdu_Mut, total read: 8617144,uniquely mapped: 3334115, read length: 75bp, layout:Single-end;
Brdu_WT, total read: 8186255,uniquely mapped: 2197645, read length: 75bp, layout:Single-end;
input_Mcm-Mut-1, total read: 7680614,uniquely mapped: 4335142, read length: 75bp, layout:Single-end;
input_Mcm-Mut-2, total read: 5145274,uniquely mapped: 3177264, read length: 75bp, layout:Single-end;
input_Mcm-WT-1, total read: 7865174,uniquely mapped: 4778981, read length: 75bp, layout:Single-end;
input_Mcm-WT-2, total read: 4794325,uniquely mapped: 2959390, read length: 75bp, layout:Single-end;
input_Orc-Mut-1, total read: 10770700,uniquely mapped: 5129173, read length: 75bp, layout:Single-end;
input_Orc-Mut-2, total read: 6402922,uniquely mapped: 1641626, read length: 75bp, layout:Single-end;
input_Orc-WT-1, total read: 6685079,uniquely mapped: 3815223, read length: 75bp, layout:Single-end;
input_Orc-WT-2, total read: 6373003,uniquely mapped: 2638958, read length: 75bp, layout:Single-end;
Mcm-Mut-1, total read: 9033760,uniquely mapped: 2161221, read length: 75bp, layout:Single-end;
Mcm-Mut-2, total read: 5998026,uniquely mapped: 2313933, read length: 75bp, layout:Single-end;
Mcm-WT-1, total read: 7975966,uniquely mapped: 1906353, read length: 75bp, layout:Single-end;
Mcm-WT-2, total read: 5051970,uniquely mapped: 2293358, read length: 75bp, layout:Single-end;
Mnase-Mut-G1, total read: 39591802,uniquely mapped: 20515968, read length: 75bp, layout:Pair-end;
Mnase-WT-G1, total read: 36096175,uniquely mapped: 20094288, read length: 75bp, layout:Pair-end;
Orc-Mut-1, total read: 7564265,uniquely mapped: 2475223, read length: 75bp, layout:Single-end;
Orc-Mut-2, total read: 7959014,uniquely mapped: 1457456, read length: 75bp, layout:Single-end;
Orc-WT-1, total read: 6974648,uniquely mapped: 2521760, read length: 75bp, layout:Single-end;
Orc-WT-2, total read: 6141728,uniquely mapped: 2085899, read length: 75bp, layout:Single-end

Antibodies

For ORC ChIP-seq, Anti-c-myc Mouse monoclonal antibody (clone 9E10) (Cat#11667149001, Lot#37569500), Roche, was used.
For MCM ChIP-seq, Monoclonal ANTI-FLAG® M2 antibody produced in mouse, clone M2, (F3165) Sigma-Aldrich, was used.
For BrdU-IP-seq, Anti-BrdU Monoclonal Antibody (ZBU30) (Cat# 03-3900, Lot#Q1215270), Invitrogen, was used

Peak calling parameters

For ChIP-seq, raw sequencing reads were mapped against yeast reference genome sacCer2 (UCSC) using Bowtie (version 1.2.2) (parameters "--phred33-quals -v 3 -m 1 -p 24 --best --strata"). PCR duplicates were marked and removed using Picard tools MarkDuplicates (version 2.9.0-1-gf5b9f50-SNAPSHOT).
For WT and mutant ORC ChIP-seq datasets, peaks were defined with each replicate using MACS2 (version 2.1.1.20160309) (parameters "--nomodel --extsize 147 -q 0.005 -g 12.07e6").
For ATAC-seq and MNase-seq, raw sequencing reads were mapped against yeast reference genome sacCer2 (UCSC) using Bowtie2 (version 2.3.4.1) (parameters " -t -q -p 24 -N 1 -L 25 -X 2000 --no-discordant --no-mixed --un-conc").
For ATAC-seq peak calling, MACS2 (version 2.1.1.20160309) was used with the following parameters "-g 12100000 --keep-dup all -B --SPMR --nomodel --shift -75 --extsize 150 -p 0.01".

Data quality

To ensure reads are properly mapped, only uniquely mapped reads were kept. Only those peaks that were called in both replicates of a sample and did not overlap with tRNA genes (GtRNAdb) were kept for downstream analysis.

Software

Bowtie (version 1.2.2)
Picard tools MarkDuplicates (version 2.9.0-1-gf5b9f50-SNAPSHOT)
samtools (version 1.3.1)
bedtools (version 2.27.1)
Deeptools (version 3.0.1)
MACS2 (version 2.1.1.20160309)

Homer (version 4.9.1-5)
Bowtie2 (version 2.3.4.1)

Flow Cytometry

Plots

Confirm that:

☒ The axis labels state the marker and fluorochrome used (e.g. CD4-FITC).

☒ The axis scales are clearly visible. Include numbers along axes only for bottom left plot of group (a 'group' is an analysis of identical markers).

☒ All plots are contour plots with outliers or pseudocolor plots.

☒ A numerical value for number of cells or percentage (with statistics) is provided.

Methodology

Sample preparation	Yeast cells were harvested and washed with sterile double-distilled water. Permeabilization was done by incubation in 70% ethanol at 4°C for 2 hours. Cells were then washed with 50mM sodium citrate buffer. RNase digestion was performed at 50°C for 1 hour in 50mM in sodium citrate buffer containing 0.25mg/ml RNase. Subsequently, Proteinase K digestion (1mg/ml) was done at 50°C for 1 hour. Cells were stained with propidium iodide (10ug/ml) and sonicated to dissociate cell clumps before flow cytometry analysis.
Instrument	BD FACSort System
Software	Flow cytometry data was collected by CELLQuest software (v3.3) and analyzed with FlowJo (v10.0.7r2) .
Cell population abundance	No cell sorting was performed with the flow cytometer.
Gating strategy	Standard FSC against SSC gate was applied to the starting cell population, followed by PI-Area against PI-Width gate to locate singlets.

☒ Tick this box to confirm that a figure exemplifying the gating strategy is provided in the Supplementary Information.
